# Supplementary material for: Identification of novel and rare variants associated with handgrip strength using whole genome sequence data from the NHLBI Trans-Omics in Precision Medicine (TOPMed) Program
Source: PLoS One. 2021 Jul 2;16(7):e0253611. doi: 10.1371/journal.pone.0253611 (PMC8253404; doi:10.1371/journal.pone.0253611)
Supplement: S1 File — (DOCX) [file pone.0253611.s001.docx]

**Supplement: Identification of novel and rare variants associated with handgrip strength using whole genome sequence data from the NHLBI Trans-Omics in Precision Medicine (TOPMed) Program**

**Contents**

[**Supplementary Text** 1](#_Toc68522909)

[**Supplementary Figures** 9](#_Toc68522910)

[**Supplementary Tables** 14](#_Toc68522911)

# **Supplementary Text**

**The Amish Study**

The Amish Complex Disease Research Program includes a set of large community-based studies focused largely on cardiometabolic health carried out in the Old Order Amish (OOA) community of Lancaster County, Pennsylvania.[1] Over 7,000 Amish have been recruited to date. This Amish community is a founder population who immigrated to Pennsylvania from Western Europe in the early 1700’s, later expanding into other regions of the U.S.

The Amish cohort participating in the TOPMed Consortium comprises 1,120 subjects ≥ 18 years of age from large multigenerational families who were recruited for specific protocols between 2001 and 2006. Subjects have been extensively phenotyped for a range of cardiometabolic traits, including anthropometry, lipids, blood pressure, glucose and related measures, vascular imaging, and a range of other phenotypes. Handgrip strength was measured in 344 of the TOPMed subjects who participated in the Amish Longevity Study. DNA samples have been collected and serum and plasma samples biobanked. The TOPMed Program has provided WGS data to complement GWAS array data already collected in >5,000 Amish study participants. Due to their ancestral history, the OOA are enriched for rare exonic variants that arose in the population from a single founder (or small number of founders) and propagated through genetic drift. Many of these variants have large effect sizes, and identifying them can lead to new biological insights about health and disease. A major goal of the TOPMed WGS sequencing efforts is to identify functional variants that underlie some of the large effect associations observed in this unique population.

**Study Design:** cross-sectional

**Ascertainment**: Amish individuals >= 90 years old, their children/siblings/spouses, and the spouses of the children/siblings were eligible for enrollment into the Amish Longevity Study. Everyone with usable DNA was sent for TOPMed sequencing.

**Other relevant information:** Age > 90 is PHI, so the ages have been binned as 90+ and should be analyzed as 90.

**Handgrip strength definition:** Handgrip strength was assessed using a Jamar Hand Dynamometer (Sammons Preston, model #5030J1). Two trials were done for each hand.

**The Atherosclerosis Risk in Communities (ARIC) Study**

The ARIC study is a population-based prospective cohort study of cardiovascular disease sponsored by the National Heart, Lung, and Blood Institute (NHLBI). ARIC included 15,792 individuals, predominantly European American and African American, aged 45-64 years at baseline (1987-89) from four US communities. Cohort members completed three additional triennial follow-up examinations, a fifth exam in 2011-2013, a sixth exam in 2016-2017, and a seventh exam in 2018-2019. The ARIC study has been described in detail previously. [2]

**Study Design:** Prospective, longitudinal cohort study

**Sample selection:** Sequencing of DNA samples from ARIC study participants was supported by the NHLBI TOPMed Whole Genome Sequencing (WGS) Project and the NHGRI Centers for Common Disease Genetics (CCDG) program. Joint variant identification and genotype calling for ARIC samples funded by both programs and included in TOPMed freeze 8 was performed by the TOPMed Informatics Research Center (IRC). The criteria for sample selection were full consent or consent for cardiovascular disease-specific research, sufficient DNA for sequencing, and unrestricted use of DNA.

**Handgrip strength definition:** Handgrip strength was measured in the participant's preferred hand using an adjustable, handheld Jamar hydraulic grip strength dynamometer (Sammons Preston Rolyan; Chicago, IL) at examination 5 (2011-2013); handgrip strength was measured in kg and represents the best handgrip strength of two assessments spaced 15-20 seconds apart. Participants were excluded if they had bilateral surgery in the hands or wrists in the previous 3 months. Acute pain or arthritis flares were recorded and participants with acute pain or flares were asked if symptoms would prevent them from squeezing as hard as they could.

**The Cardiovascular Health Study (CHS)**

CHS is a population-based cohort study of risk factors for coronary heart disease and stroke in adults ≥65 years conducted across four field centers. [3] The original predominantly European ancestry cohort of 5,201 persons was recruited in 1989-1990 from random samples of the Medicare eligibility lists; subsequently, an additional predominantly African-American cohort of 687 persons was enrolled for a total sample of 5,888. CHS was approved by institutional review committees at each field center and individuals in the present analysis had available DNA and gave informed consent including consent to use of genetic information for the study of cardiovascular disease.

**Study Design:** longitudinal

**TOPMed Sample Selection:** CHS participants were selected for inclusion in the TOPMed sequencing program if they had appropriate consent, available DNA, and met any of the following criteria:

1. had an adjudicated idiopathic VTE event during follow-up
2. were African American,
3. had an incident MI or definite fatal coronary heart disease (CHD) event during follow-up,
4. had a probable fatal CHD event during follow-up,
5. had an incident stroke during follow-up,
6. had a prevalent MI or stroke at baseline, or
7. were part of a random sample of “healthy elderly” participants who survived free of an MI or stroke.

Selection of participants proceeded in a hierarchical manner from criterion a) to g) without replacement. Each participant is thus assigned to one, and only one, of the seven groups. Follow-up for MI, stroke, and CHD events occurred from baseline in 1989-90 or 1992-1993 through June 30, 2014. Follow-up for VTE events occurred from baseline (1989-90 or 1992-1993) through December 31, 2001.

**Handgrip strength** **definition:** Handgrip strength was measured using a handheld Jamar Dynamometer (Asimow Engineering Co., Los Angeles, CA) at each annual study visit from 1989-1998, with the exception of the study visit in 1990 (total of 9 annual measurements). Three trials were performed in each hand.

Participants were excluded from testing if they reported either of the following conditions:

1. Acute flare-up of wrist/hand; for example, arthritis, tendinitis or carpal tunnel syndrome.

2. Less than 13 weeks after surgery for fusion, arthroplasty, tendon repair or synovectomy of the upper extremity.

**The Framingham Heart Study (FHS)**

The FHS is a large-scale prospective, population-based and longitudinal study that was initiated in 1948 to better understand the determinants of cardiovascular disease. At study initiation, participants were residents of the town of Framingham (MA), almost entirely of European descent and free of overt symptoms of cardiovascular disease, heart attack or stroke. The FHS is composed of three generations of participants. The first generation (Original cohort/Gen1) has been followed since 1948 and included 5,209 participants. Survivors are still invited to participate in examinations every two years.[4] The second generation (Offspring cohort/Gen2) has been followed since 1971 and is comprised of 5,124 offspring of the original cohort and spouses of these offspring, including 3,514 biological offspring. They have attended examinations every 4 to 8 years. [5] The third generation (Gen3) was enrolled in 2002 and included 4,095 children from the largest families of the Offspring cohort. They have attended three examinations 4 years apart. [6] All cohorts are still under active surveillance for cardiovascular events, stroke, and dementia.

**Design:** Family and case-control, longitudinal study

**Handgrip strength** **definition**: Handgrip strength was measured with a Jamar dynamometer with three trials performed in each hand, and the maximum of the six trials for each participant was selected.

**The Hypertension Genetic Epidemiology Network (HyperGEN)**

The HyperGEN study is one of the four networks in the Family Blood Pressure Program (FBPP) supported by the National Heart, Lung, and Blood Institute to identify genetic contributors to hypertension. [7] HyperGEN is a family-based study with a sib-pair design. Hypertensive African American sibships were recruited from population-based cohorts in Forsyth County, NC, and from the community-at-large in Birmingham, AL, from 1995 to 2000. Sibling pairs with onset of hypertension before age 60 were recruited in the first phase. The study was later extended to other siblings and the offspring of the hypertensive probands who were unmedicated adults. Participants were ineligible for the handgrip strength study if they had had a significant cardiovascular event (myocardial infarction, stroke, coronary artery bypass surgery, angioplasty, unstable angina) in the previous three months. The study was approved by the Institutional Review Boards of the participating organizations. All HyperGEN participants provided informed consent for use of samples and data for subsequent analyses.

**Handgrip strength** **definition:** Handgrip strength was measured with a dynamometer with two trials performed using the non-dominant hand; the average of the two maximum handgrip strength pressures was computed.

**The Women’s Health Initiative (WHI)**

The Women’s Health Initiative (WHI) is a long-term, prospective, multi-center cohort study investigating post-menopausal women’s health in the US. WHI was funded by the National Institutes of Health and the National Heart, Lung, and Blood Institute to study strategies to prevent heart disease, breast cancer, colon cancer, and osteoporotic fractures in women 50-79 years of age.[8] WHI involves 161,808 women recruited between 1993 and 1998 at 40 centers across the US. The study consists of two parts: the WHI Clinical Trial which was a randomized clinical trial of hormone therapy, dietary modification, and calcium/Vitamin D supplementation, and the WHI Observational Study, which focused on many of the inequities in women’s health research and provided practical information about incidence, risk factors, and interventions related to heart disease, cancer, and osteoporotic fractures.

**Study Design:** longitudinal

**Ascertainment*:*** Outcomes were ascertained as follows:

All WHI clinical outcomes were identified by self-report on forms administered semi-annually to Clinical Trial (CT) participants and annually to Observational Study (OS) participants. Those participants who reported a potential WHI-defined outcome were then contacted by mail or phone and a form was completed to obtain more specific information on newly diagnosed health conditions or recent procedures. Investigation of potential WHI clinical outcomes depended on the type of outcome, the participant’s study component, and whether or not the outcome was a first versus a recurrent event. A documentation set was defined for each type of WHI clinical outcome, and these medical records were used for adjudication.

*Local Adjudication*

Based on specific adjudication criteria, a physician adjudicator at the local Clinical Center either confirmed or denied a potential clinical outcome. Data on confirmed WHI outcomes were entered on outcomes-specific [forms](https://www.whi.org/researchers/data/Pages/WHI-Forms.aspx) for cardiovascular disease, cancer, fracture, hysterectomy, venous thromboembolic disease, as well as hospitalizations and deaths.

*Central Adjudication*

WHI Clinical Centers were asked to send selected locally adjudicated case packets to the Clinical Coordinating Center for central adjudication, depending on the type of outcome and the participant’s study component. Centrally-trained cardiovascular physician adjudicators (and neurologists, in the case of stroke outcomes) reviewed and adjudicated cardiovascular and death outcomes. The five primary cancers (breast, colon, rectum, endometrium, and ovary) were coded centrally by tumor registry coders. All hip fractures were centrally adjudicated at the WHI Bone Density Center.

**TOPMed sample selection**: 11,100WGS

- Incident stroke (~n=4000 ischemic and 900 hemorrhagic)

- Extremes of blood pressure (n=1000-2000 with high BP and 1000-2000 with low BP)

- Normotensive comparison group with no stroke (n=2000)

- VTE cases (~n=1100)

- WHI controls drawn from stroke study controls.

Participants who were in the dataset used for handgrip strength analysis came from several components of the WHI that have been sequenced in TOPMED. This is illustrated in the Venn diagram below.


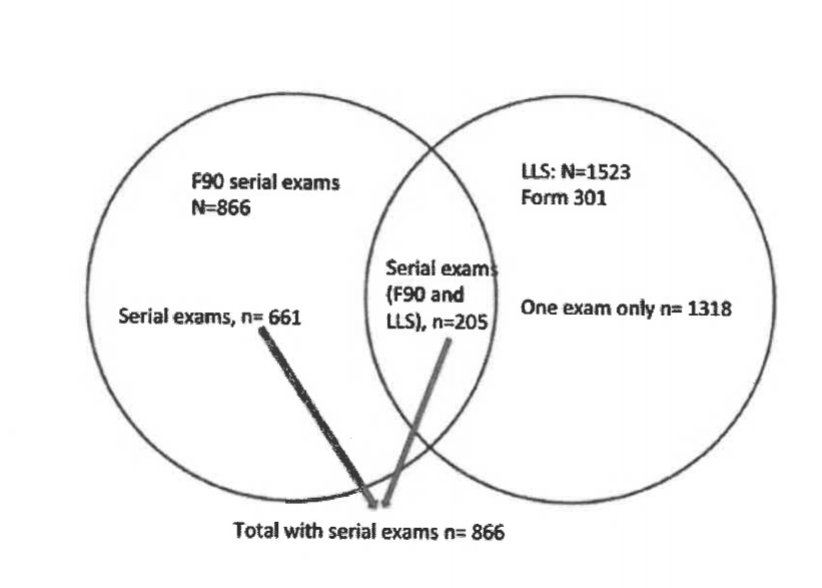


Of the total N=2,184 participants in the dataset, N=1,318 have a single handgrip strength exam and N=866 have serial (2 or more) handgrip strength exams.

1. **1,523 women from the Long Life Study** (a sub-study of WHI which has N = 7,785 total in LLS). These women had a home visit in 2012-2013 during which handgrip strength was measured. They were in the Medical Records Cohort (MRC) in Extension 2 (2010-2015) of WHI, which includes all WHI Hormone Trial participants and all Black/African-American and Hispanic/Latino participants from the Clinical trials or Observational Study. Of the N=1,523 women who are in TOPMED, N=1,318 had only one handgrip strength exam in the LLS. The remaining N=205 women were a subsample who had all been in the hormone trials and had previously had handgrip strength exams at WHI baseline (1994-1998), years 3, 6 and 9, and thus had serial handgrip strength exams (though not all these women had all of these exams, but they each had 2 or more).
2. a **subset of N=866 women** who had been in the hormone trials (and are in TOPMED) who had handgrip strength measured serially, at WHI baseline, years 3, 6 and 9

**Handgrip strength** **definition:** Handgrip strength was measured using a handheld dynamometer- Jamar hand dynamometer, Lafayette Instruments, Lafayette, IN. Two trials were performed in the dominant hand.

**References**

1. Sorkin J, Post W, Pollin TI, O'Connell JR, Mitchell BD, Shuldiner AR. Exploring the genetics of longevity in the Old Order Amish. Mech Ageing Dev. 2005;126: 347-350. doi: S0047-6374(04)00210-6 [pii].

2. [Anonymous]. The Atherosclerosis Risk in Communities (ARIC) Study: design and objectives. The ARIC investigators. Am J Epidemiol. 1989;129: 687-702.

3. Fried LP, Borhani NO, Enright P, Furberg CD, Gardin JM, Kronmal RA, et al. The Cardiovascular Health Study: design and rationale. Ann Epidemiol. 1991;1: 263-276. doi: 1047-2797(91)90005-W [pii].

4. Dawber TR, Kannel WB. The Framingham study. An epidemiological approach to coronary heart disease. Circulation. 1966;34: 553-555.

5. Feinleib M, Kannel WB, Garrison RJ, McNamara PM, Castelli WP. The Framingham Offspring Study. Design and preliminary data. Prev Med. 1975;4: 518-525.

6. Splansky GL, Corey D, Yang Q, Atwood LD, Cupples LA, Benjamin EJ, et al. The Third Generation Cohort of the National Heart, Lung, and Blood Institute's Framingham Heart Study: design, recruitment, and initial examination. Am J Epidemiol. 2007;165: 1328-1335. doi: kwm021 [pii].

7. Williams RR, Rao DC, Ellison RC, Arnett DK, Heiss G, Oberman A, et al. NHLBI family blood pressure program: methodology and recruitment in the HyperGEN network. Hypertension genetic epidemiology network. Ann Epidemiol. 2000;10: 389-400. doi: S1047279700000636 [pii].

8. [Anonymous]. Design of the Women's Health Initiative clinical trial and observational study. The Women's Health Initiative Study Group. Control Clin Trials. 1998;19: 61-109. doi: S0197245697000780 [pii].

9. Willems SM, Wright DJ, Day FR, Trajanoska K, Joshi PK, Morris JA, et al. Large-scale GWAS identifies multiple loci for hand grip strength providing biological insights into muscular fitness. Nat Commun. 2017;8: 16015. doi: 10.1038/ncomms16015 [doi].

# **Supplementary Figures**

**S1 Fig.** Manhattan-plot of the pooled whole genome sequence association of *MEAN* handgrip strength in 13,552 individuals from TOPMed.


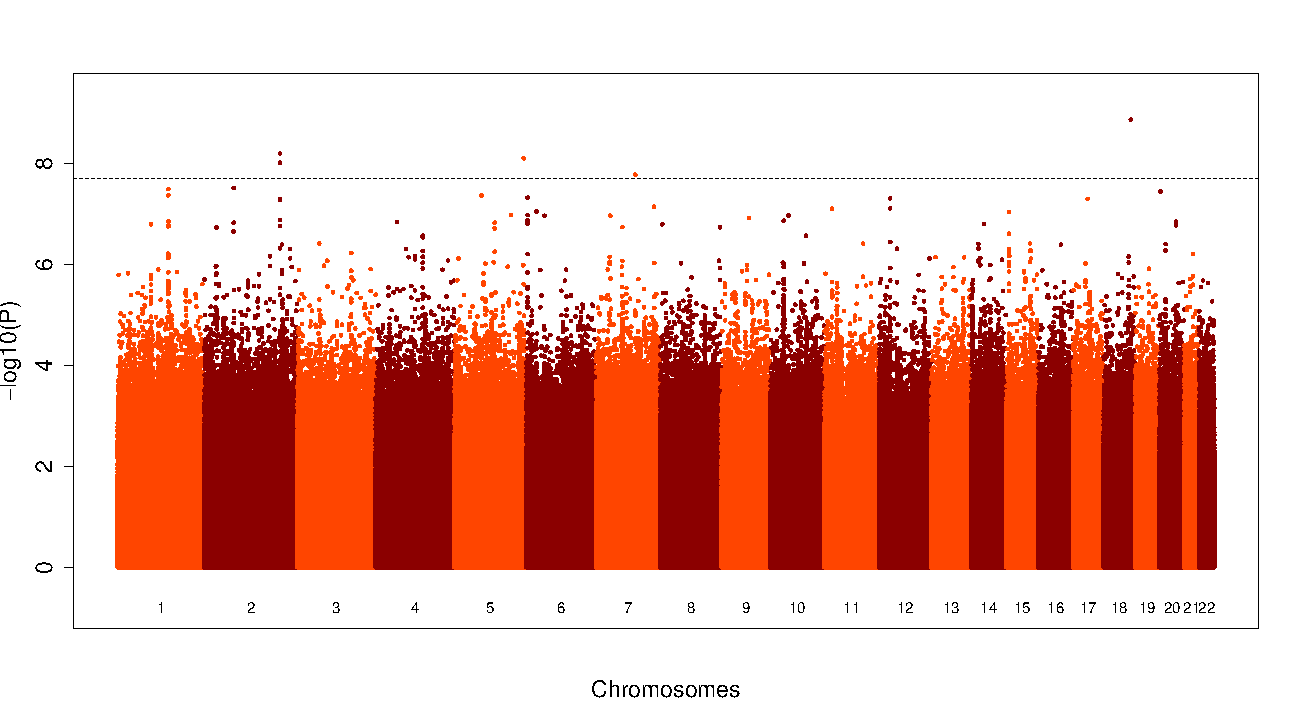


The –log10(*P*)-value for each single nucleotide variant on the y-axis is plotted against the build 38 genomic position on the x-axis (chromosomal coordinate). The dashed horizontal line indicates the genome-wide significance threshold of *P* = 2$\times$10^-8^.

**S2 Fig.** Quantile-Quantile (QQ) plot of the pooled whole genome sequence association of *MEAN* handgrip strength in 13,552 individuals from TOPMed.


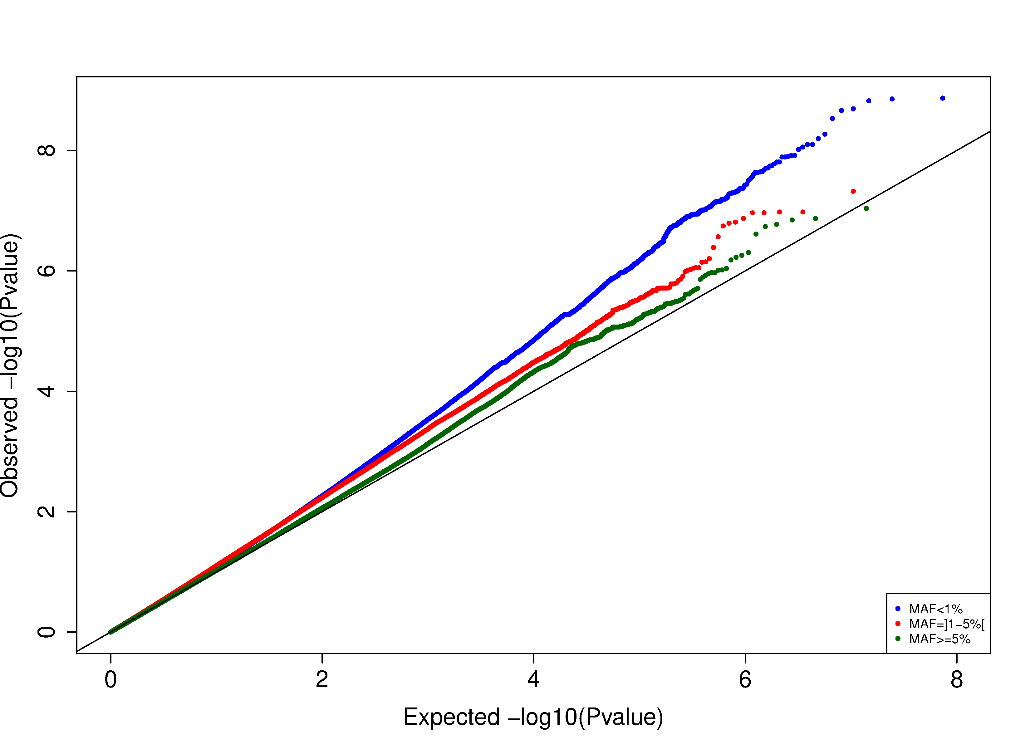


The dots represent the distribution of observed ordered –log10(*P*) against the theoretical model distribution of expected ordered –log10(*P*). The solid black line represents the theoretical model distribution of expected –log10(*P*) under the null distribution. The genomic inflation factor (λ_GC_=1.08) is defined as the ratio of the median of the empirically observed distribution of the test statistic to the expected median, thus quantifying the extent of the inflation and the excess false positive rate. Association results were stratified by minor allele frequency (MAF) of single nucleotide variants: MAF ≥ 5% are indicated in green (λ_GC_=1.03); 5% > MAF ≥ 1% are indicated in red (λ_GC_=1.12); MAF<1% are indicated in blue (λ_GC_=1.09).

**S3 Fig.** Two-by-two correlations of –log10(*P*) from the association results of the three models of handgrip strength outcome (*MEAN*, *ALL*, *ONE*) for variants with a MAF greater or equal to 0.001


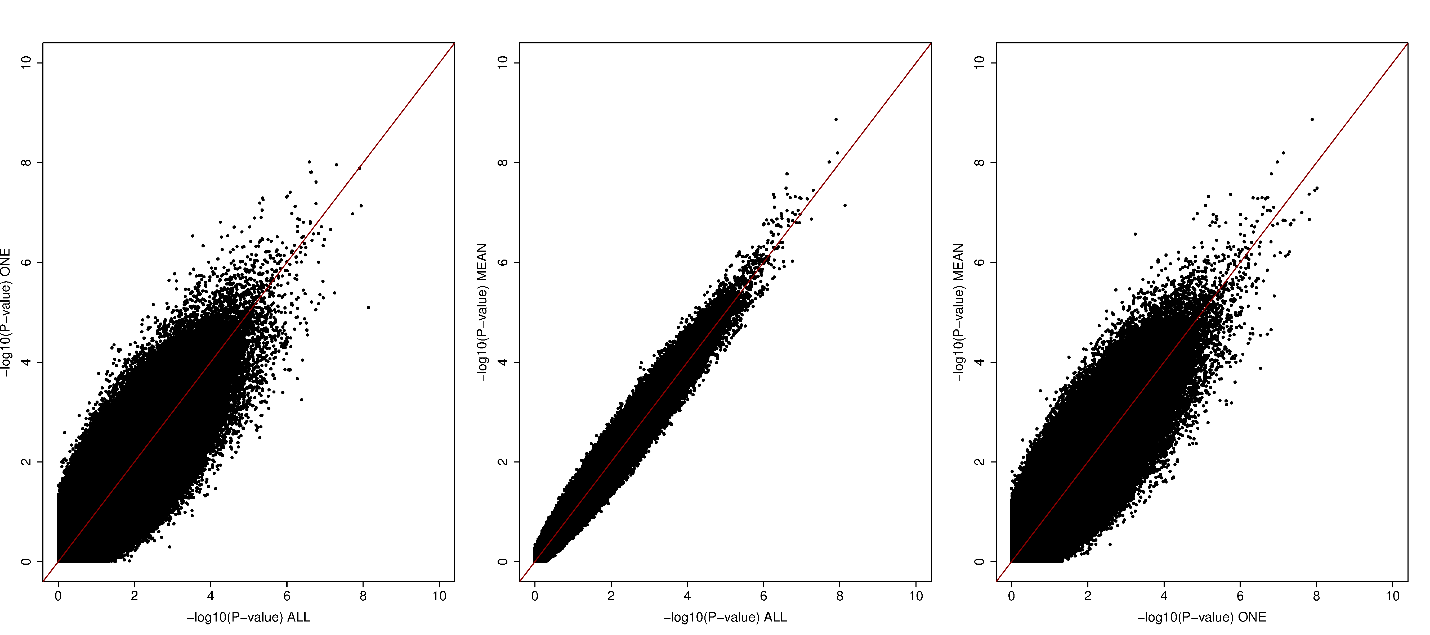


Correlation of –log10(*P*) were equal to 0.85 (*ALL* vs *ONE*), 0.98 (*ALL* vs *MEAN*) and 0.87 (*ONE* vs *MEAN*)

**S4 Fig.** Two-by-two correlations of effect sizes from the association results of the three models of handgrip strength outcome (*MEAN*, *ALL*, *ONE*) for variants with a MAF greater or equal to 0.001


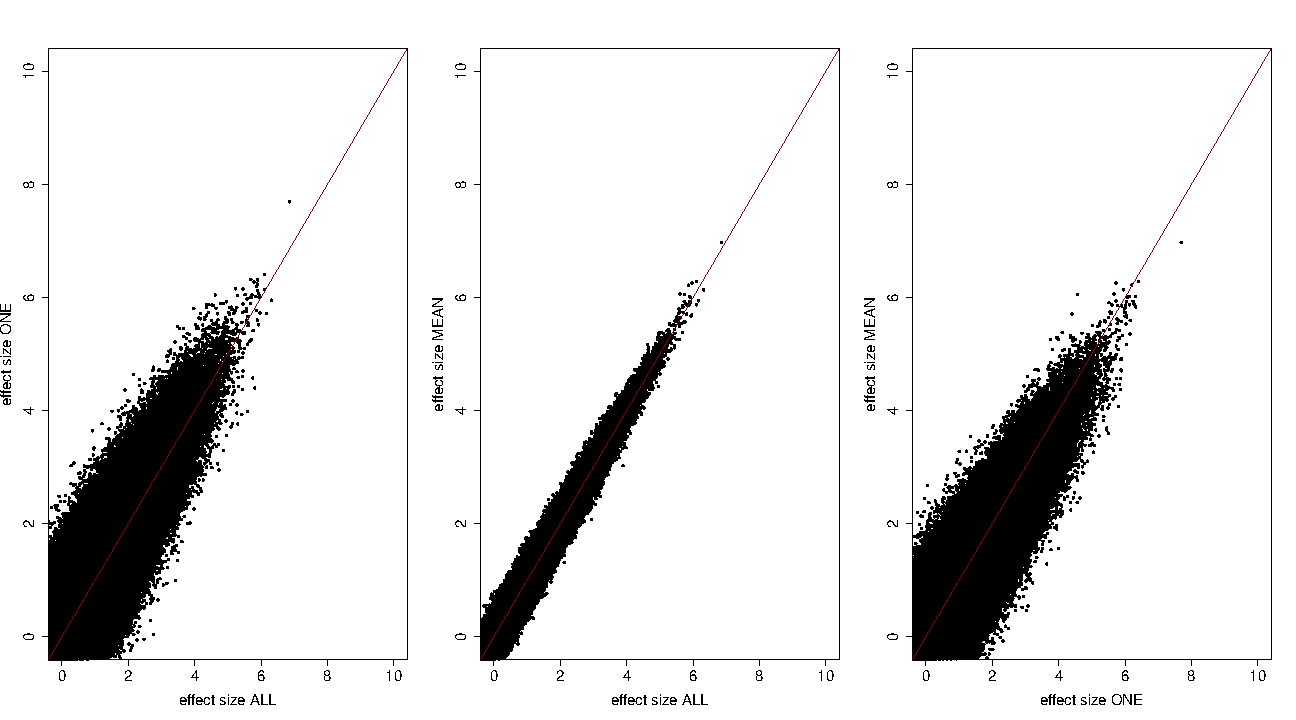


Correlation of effect sizes were equal to 0.93 (*ALL* vs *ONE*), 0.99 (*ALL* vs *MEAN*) and 0.94 (*ONE* vs *MEAN*)

**S5 Fig.** Comparison of effect sizes and effect allele frequency between TOPMed *MEAN* analyses and UKBB handgrip strength GWAS (Willems et al, 2017)

**
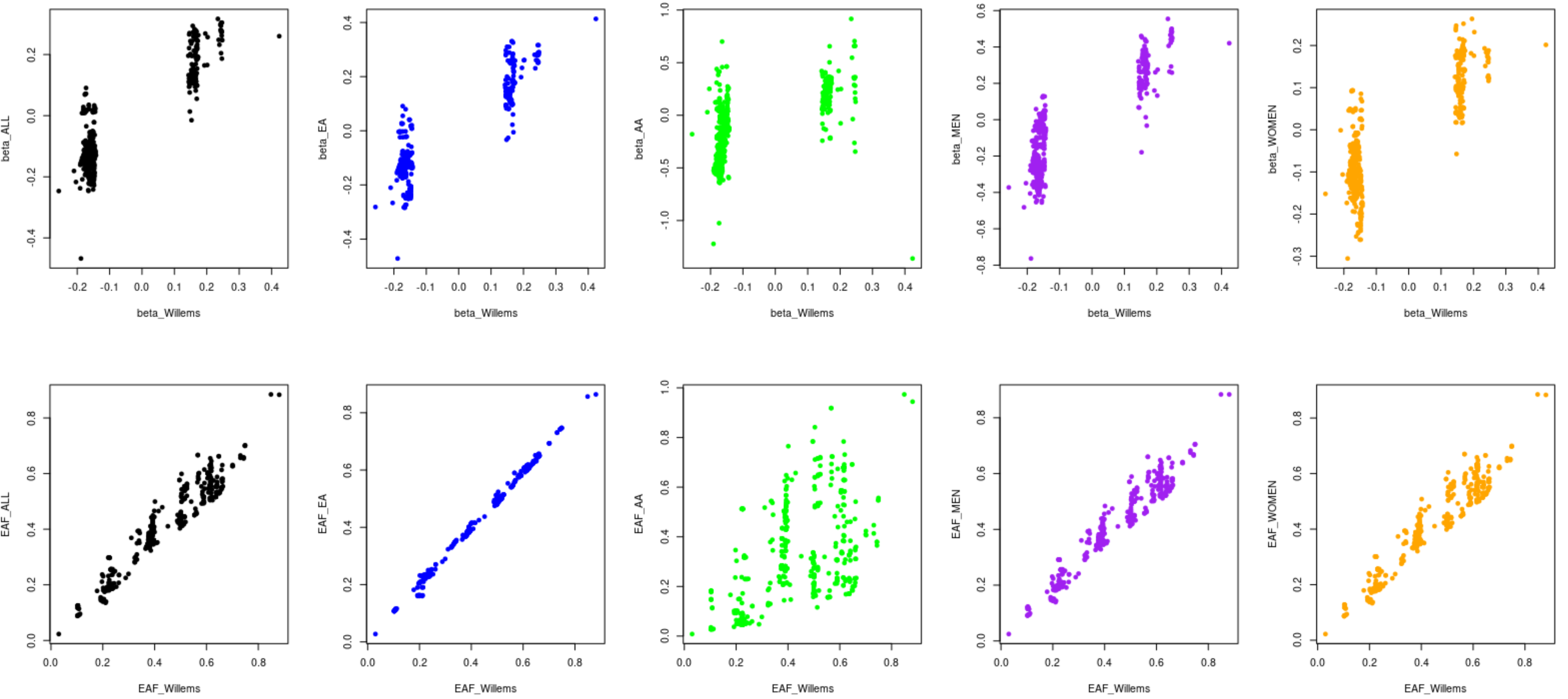
**

EAF: Effect Allele Frequency; EA: European-American; AA: African-American

# **Supplementary Tables**

**S1 Table.** Distribution of the study-reported population groups by study for the 13,552 TOPMed participants included in the main pooled whole genome sequence association analysis of *MEAN* handgrip strength

| **Study** | **EA** | **AA** | **Hispanic or Latino** | **Asian or Pacific-Islander** | **American-Indian or Alaskan-Native** | **Other** | **Total** |
| --- | --- | --- | --- | --- | --- | --- | --- |
| ARIC | 2,588 | 457 | 0 | 0 | 0 | 0 | 3,045 |
| Amish | 344 | 0 | 0 | 0 | 0 | 0 | 344 |
| CHS | 2,789 | 536 | 0 | 2 | 4 | 8 | 3,339 |
| FHS | 3,083 | 0 | 0 | 0 | 0 | 0 | 3,083 |
| HyperGEN | 0 | 1,696 | 0 | 0 | 0 | 1 | 1,697 |
| WHI | 1,459 | 456 | 107 | 15 | 4 | 3 | 2,044 |
| Total | 10,263 | 3,145 | 107 | 17 | 8 | 12 | 13,552 |

EA: White/European American; AA: Black/African American

| **Number of exams** | **ARIC** | **Amish** | **CHS** | **FHS** | **HyperGEN** | **WHI** | **Total** |
| --- | --- | --- | --- | --- | --- | --- | --- |
| 1 | 3,045 | 344 | 135 | 1,467 | 1,697 | 1,217 | 7,905 |
| 2 | 0 | 0 | 135 | 692 | 0 | 65 | 892 |
| 3 | 0 | 0 | 207 | 924 | 0 | 152 | 1,283 |
| 4 | 0 | 0 | 271 | 0 | 0 | 426 | 697 |
| 5 | 0 | 0 | 301 | 0 | 0 | 184 | 485 |
| 6 | 0 | 0 | 477 | 0 | 0 | 0 | 477 |
| 7 | 0 | 0 | 278 | 0 | 0 | 0 | 278 |
| 8 | 0 | 0 | 500 | 0 | 0 | 0 | 500 |
| 9 | 0 | 0 | 1,035 | 0 | 0 | 0 | 1.035 |
| **Total** | 3,045 | 344 | 3,339 | 3,083 | 1,697 | 2,044 | 13,552 |

**S2 Table.** Distribution of the number of exams with handgrip strength information by study for the 13,552 TOPMed participants

**S3 Table.** Description of the 13,552 TOPMed participants included in the main pooled whole genome sequence association analysis of *MEAN* handgrip strength and the 8,408 UK Biobank participants used for replication, by study and stratified by sex

|  | **TOTAL (N=13,552)** | | | **MEN (N=4,878)** | | | **WOMEN (N=8,674)** | | |
| --- | --- | --- | --- | --- | --- | --- | --- | --- | --- |
| **Study** | **N** | **Age, mean years (SD)** | **Handgrip (kg)** | **N** | **Age, mean years (SD)** | **Handgrip (kg)** | **N** | **Age, mean years (SD)** | **Handgrip (kg)** |
| Amish | 344 | 66 (11) | 30.4 (10.7) | 160 | 67 (11) | 37.1 (9.7) | 184 | 65 (11) | 24.7 (7.7) |
| ARIC | 3,045 | 75 (5) | 28.8 (10.3) | 1,271 | 76 (5) | 37.1 (9.0) | 1,774 | 75 (5) | 22.9 (6.2) |
| CHS | 3,339 | 76 (5) | 29.4 (9.6) | 1,398 | 77 (5) | 37.9 (7.8) | 1,941 | 76 (5) | 23.3 (5.2) |
| FHS | 3,083 | 60 (14) | 33.1 (12.1) | 1,421 | 61 (13) | 42.8 (9.9) | 1,662 | 60 (14) | 24.8 (6.4) |
| HyperGEN | 1,697 | 47 (13) | 34.2 (12.7) | 628 | 46 (13) | 45.6 (11.9) | 1,069 | 47 (13) | 27.5 (7.1) |
| WHI | 2,044 | 77 (6) | 20.9 (6.2) | -- | -- | -- | 2,044 | 77 (6) | 20.9 (6.2) |
| **Total** | 13,552 | 69 (14) | 29.5 (11.2) | 4,878 | 68 (14) | 40.1 (9.9) | 8,674 | 69 (14) | 23.5 (6.5) |
| **UKBB AA*** | 8,408 | 52 (8) | 34.0 (11.7) | 3,548 | 52 (8) | 43.2 (10.4) | 4,860 | 52 (8) | 27.3 (7.1) |

Mean age was calculated across all exams for which handgrip strength was available for the *MEAN* analysis

*We identified UKBB African-ancestry participants using the following six self-reported ancestries: "Caribbean", "African", "Black or Black British", "Any other Black background", "White and Black African" and "White and Black Caribbean".

**S4 Table.** Description of the 13,552 TOPMed participants included in the main pooled whole genome sequence association analysis of *MEAN* handgrip strength and the 8,408 UK Biobank participants used for replication, by study, and stratified by study-reported population group for the two largest population groups

|  | **TOTAL (N=13,552)** | | | **EA (N=10,263)** | | | **AA (N=3,145)** | | |
| --- | --- | --- | --- | --- | --- | --- | --- | --- | --- |
| **Study** | **N** | **Age, mean years (SD)** | **Handgrip (kg)** | **N** | **Age, mean years (SD)** | **Handgrip (kg)** | **N** | **Age, mean years (SD)** | **Handgrip (kg)** |
| Amish | 344 | 66 (11) | 30.4 (10.7) | 344 | 66 (11) | 30.4 (10.7) | -- | -- | -- |
| ARIC | 3,045 | 75 (5) | 28.8 (10.3) | 2,588 | 76 (5) | 28.2 (10.2) | 457 | 74 (5) | 32.3 (9.8) |
| CHS | 3,339 | 76 (5) | 29.4 (9.6) | 2,789 | 76 (5) | 29.0 (9.6) | 536 | 76 (5) | 31.2 (9.8) |
| FHS | 3,083 | 60 (14) | 33.1 (12.1) | 3,083 | 60 (14) | 33.1 (12.1) | -- | -- | -- |
| HyperGEN | 1,697 | 47 (13) | 34.2 (12.7) | -- | -- | -- | 1,696 | 47 (13) | 34.2 (12.7) |
| WHI | 2,044 | 77 (6) | 20.9 (6.2) | 1,459 | 77 (6) | 20.7 (5.9) | 456 | 76 (6) | 21.7 (7.0) |
| **Total** | 13,552 | 69 (14) | 29.5 (11.2) | 10,263 | 71 (11) | 28.9 (10.9) | 3,145 | 60 (18) | 31.6 (11.9) |
| **UKBB AA*** | 8,408 | 52 (8) | 34.0 (11.7) | -- | -- | -- | 8,408 | 52 (8) | 34.0 (11.7) |

Mean age was calculated across all exams for which handgrip strength was available for the *MEAN* analysis

--: no participants in this category

*We identified UKBB African-ancestry participants using the following six self-reported ancestries: "Caribbean", "African", "Black or Black British", "Any other Black background", "White and Black African" and "White and Black Caribbean".

**S5 Table.** Main association results from the WGS association analysis of *MEAN* handgrip strength (*P*<2×10^-8^ in at least one analysis) and stratified by sex

|  |  |  |  |  | **TOTAL (N=13,552)** | | | | **MEN (N=4,878)** | | | | **WOMEN (N=8,674)** | | |  |  |  |
| --- | --- | --- | --- | --- | --- | --- | --- | --- | --- | --- | --- | --- | --- | --- | --- | --- | --- | --- |
| **Chr** | **Pos (build 38)** | **rsid** | **Ref** | **Alt** | **EAF** | **Beta** | ***P*** | **EAF** | | **Beta** | ***P*** | **EAF** | | **Beta** | ***P*** | ***P*_het_** | **Gene** | |
| 2 | 198,070,149 | rs74688411 | A | G | 0.004 | 3.84 | 6.4E-09 | 0.004 | | 6.27 | 3.7E-05 | 0.005 | | 2.52 | 9.4E-05 | 0.02 | *PLCL1* | |
| 5 | 76,919,524 | rs577776684 | C | T | 0.003 | -2.84 | 5.1E-04 | 0.002 | | -12.22 | 1.5E-08 | 0.004 | | -0.36 | 6.4E-01 | 1.9E-07 | *S100Z* | |
| 5 | 174,630,802 | rs377692678 | G | A | 0.0008 | -8.66 | 8.0E-09 | 0.001 | | -9.51 | 8.3E-04 | 0.001 | | -7.48 | 6.3E-05 | 0.55 | *intergenic* | |
| 7 | 95,524,137 | rs544430450 | G | A | 0.001 | -6.66 | 1.7E-08 | 0.001 | | -5.77 | 2.9E-02 | 0.001 | | -6.37 | 1.6E-07 | 0.84 | *ASB4* | |
| 8 | 30,273,968 | rs2958754 | G | A | 0.023 | -1.28 | 1.0E-05 | 0.019 | | -0.10 | 8.8E-01 | 0.026 | | -1.63 | 1.3E-08 | 0.04 | *intergenic* | |
| 10 | 85,774,936 | rs569475444 | G | C | 0.005 | -1.76 | 5.5E-03 | 0.005 | | -8.09 | 9.2E-09 | 0.005 | | 0.99 | 1.2E-01 | 3.7E-09 | *GRID1* | |
| 10 | 119,692,415 | rs189542078 | C | T | 0.001 | 5.36 | 1.0E-05 | 0.001 | | -1.50 | 6.3E-01 | 0.001 | | 6.57 | 1.7E-08 | 0.01 | *intergenic* | |
| 11 | 113,977,732 | rs182799368 | G | T | 0.003 | -3.13 | 4.2E-05 | 0.003 | | -9.18 | 1.2E-08 | 0.003 | | -0.11 | 8.9E-01 | 3.7E-07 | *HTR3A* | |
| 14 | 96,043,941 | rs143569685 | T | A | 0.003 | -3.57 | 8.1E-07 | 0.003 | | -2.10 | 2.6E-01 | 0.004 | | -3.92 | 1.3E-08 | 0.36 | *C14orf132* | |
| 18 | 71,404,509 | rs185725127 | A | C | 0.001 | -6.92 | 1.4E-09 | 0.001 | | -7.26 | 8.9E-03 | 0.001 | | -6.40 | 2.8E-08 | 0.77 | *intergenic* | |

EAF: Effect allele Frequency; Alt: Alternate (Effect) allele; Pos: Positions in build GRCh38; Beta: effect size, unit (kg); P_het_: heterogeneity P-value from the meta-analysis of MEN and WOMEN accounting for the correlation (r=0.016) of effect sizes between men and women statistics due to relatedness.

**S6 Table.** Genetic variants with MAF greater or equal to 0.001 and passing the genome-wide threshold (*P*<2×10^-8^) in at least one handgrip strength outcome model (*ALL*, *ONE*, *MEAN*)

|  |  |  |  |  | ***ALL* (N=36,872)** | | | ***ONE* (N=13,552)** | | | ***MEAN* (N=13,552)** | | |
| --- | --- | --- | --- | --- | --- | --- | --- | --- | --- | --- | --- | --- | --- |
| **Chr** | **Pos (build 38)** | **Gene** | **Ref** | **Alt** | **EAF** | **Score** | ***P*** | **EAF** | **Score** | ***P*** | **EAF** | **Score** | ***P*** |
| 1 | 158,918,033 | *intergenic* | T | G | 0.003 | -6.74 | 2.6E-07 | 0.003 | -7.35 | 9.7E-09 | 0.003 | -7.42 | 3.2E-08 |
| 2 | 198,070,149 | *PLCL1* | A | G | 0.004 | 8.57 | 1.1E-08 | 0.004 | 7.80 | 7.3E-08 | 0.004 | 8.78 | 6.4E-09 |
| 7 | 95,524,137 | *ASB4* | G | A | 0.001 | -4.27 | 2.4E-07 | 0.001 | -4.23 | 1.5E-07 | 0.001 | -4.78 | 1.7E-08 |
| 7 | 146,271,555 | *CNTNAP2* | C | T | 0.003 | -7.00 | 7.3E-09 | 0.003 | -5.23 | 8.1E-06 | 0.003 | -6.60 | 7.2E-08 |
| 18 | 71,404,509 | *intergenic* | A | C | 0.001 | -4.83 | 1.3E-08 | 0.001 | -4.77 | 1.3E-08 | 0.001 | -5.30 | 1.4E-09 |
| 20 | 2,336,684 | *TGM3* | C | T | 0.001 | 4.33 | 5.0E-08 | 0.001 | 4.25 | 1.1E-08 | 0.001 | 4.35 | 3.6E-08 |

EAF: Effect allele Frequency; Alt: Alternate (Effect) allele; Pos: Positions in build GRCh38

**S7 Table.** Effective sample size calculated when leveraging 36,872 handgrip strength observations from 13,552 TOPMed participants

| **Study** | **N** | **N_eff_^*^** | **N_obs_** |
| --- | --- | --- | --- |
| ARIC | 3,045 | 3,045 | 3,045 |
| Amish | 344 | 344 | 344 |
| CHS | 3,339 | 3,825 | 21,736 |
| FHS_Offsp | 1,923 | 2,196 | 4,463 |
| FHS_gen3 | 1,160 | 1,160 | 1,160 |
| HYperGEN | 1,697 | 1,697 | 1,697 |
| WHI | 2,044 | 2,285 | 4,427 |
| **Total** | 13,552 | 14,552 | 36,872 |

$*Neff=\sum neff$;$neff=\frac{n}{1+(n-1)\times\rho}$ where ρ is the mean of the maximum correlation observed across exams (study-specific) in men and women; n is the number of handgrip strength observations for the participant; ρ was equal to 0.84, 0.76, and 0.69 in CHS, FHS, and WHI respectively.

The effective sample sizes using variance ratios between analyses leveraging multiple observations (*ALL* or *MEAN*) versus one (*ONE*) were equal to 15,095 (*ALL* vs *ONE*) and 15,224 (*MEAN* vs *ONE*).

**S8 Table.** Look-up of the six low-frequency or common index variants associated with handgrip strength in TOPMed association analysis of *MEAN* handgrip strength in the UKBB African-ancestry participants

|  |  |  |  |  |  | **UKBB African-ancestry (N=8,408)** | | | | **UKBB African-ancestry Males (N=3,548)** | | | | **UKBB African-ancestry Females (N=4,860)** | | | |
| --- | --- | --- | --- | --- | --- | --- | --- | --- | --- | --- | --- | --- | --- | --- | --- | --- | --- |
| **Chr** | **Pos** | **rsid** | **Ref** | **Alt** | **Info** | **EAF UKBB** | **EAF TOPMed** | **Beta** | **P** | **EAF UKBB** | **EAF TOPMed** | **Beta** | ***P*** | **EAF UKBB** | **EAF TOPMed** | **Beta** | ***P*** |
| 2 | 198,070,149 | rs74688411 | A | G | 0.87 | 0.016 | 0.018 | 0.46 | 0.37 | 0.015 | 0.018 | 0.29 | 0.77 | 0.017 | 0.018 | 0.58 | 0.30 |
| 5 | 76,919,524 | rs577776684 | C | T | 0.64 | 0.004 | 0.013 | -1.32 | 0.27 | 0.006 | 0.011 | -1.49 | 0.41 | 0.003 | 0.014 | -1.25 | 0.44 |
| 8 | 30,273,968 | rs2958754 | G | A | 0.96 | 0.090 | 0.096 | -0.10 | 0.66 | 0.094 | 0.085 | 0.23 | 0.57 | 0.087 | 0.101 | -0.37 | 0.13 |
| 10 | 85,774,936 | rs569475444 | G | C | 0.88 | 0.006 | 0.022 | -0.89 | 0.29 | 0.007 | 0.024 | -2.52 | 0.09 | 0.006 | 0.021 | 0.24 | 0.80 |
| 11 | 113,977,732 | rs182799368 | G | T | 0.90 | 0.012 | 0.013 | -0.38 | 0.53 | 0.013 | 0.016 | -1.13 | 0.27 | 0.011 | 0.012 | 0.32 | 0.63 |
| 11 | 113,987,110 | rs186735451 | T | C | 0.88 | 0.012 | 0.014 | -0.33 | 0.58 | 0.013 | 0.016 | -0.91 | 0.38 | 0.011 | 0.012 | 0.25 | 0.71 |

EAF: Effect allele Frequency; Alt: Alternate (Effect) allele; Pos: Positions in build GRCh38; Beta: effect size, unit (kg); Info: Imputation quality

**S9 Table.** Association results from the whole genome sequence association analysis of *MEAN* handgrip strength for the 16 lead variants reported in the UK Biobank and CHARGE handgrip strength GWAS [9]

|  |  |  | **UKBB (N=142,343)** | | | | | **TOPMed** | | | | | | | | | | | | |
| --- | --- | --- | --- | --- | --- | --- | --- | --- | --- | --- | --- | --- | --- | --- | --- | --- | --- | --- | --- | --- |
|  |  |  |  |  |  |  |  | **TOTAL (N=13,552)** | | | **EA (N=10,263)** | | | **AA (N=3,145)** | | | **MEN (N=4,878)** | | **WOMEN (N=8,674)** | |
| **Chr** | **Pos** | **rsid** | **Ref** | **Alt** | **EAF** | **B** | ***P*** | **B** | ***P*** | **EAF** | | **B** | ***P*** | **EAF** | **B** | ***P*** | **B** | ***P*** | **B** | ***P*** |
| 1 | 10,573,188 | rs6687430 | G | A | 0.54 | -0.15 | 7.6E-09 | -0.21 | 0.006 | 0.55 | | -0.21 | 0.01 | 0.32 | -0.26 | 0.20 | -0.42 | 0.008 | -0.11 | 0.16 |
| 1 | 53,646,060 | rs4926611 | T | C | 0.64 | 0.17 | 1.3E-10 | 0.19 | 0.02 | 0.62 | | 0.18 | 0.04 | 0.17 | 0.35 | 0.18 | 0.26 | 0.13 | 0.14 | 0.10 |
| 2 | 40,127,548 | rs2110927 | C | T | 0.73 | -0.16 | 4.4E-08 | -0.05 | 0.53 | 0.73 | | -0.02 | 0.83 | 0.41 | -0.18 | 0.36 | -0.02 | 0.91 | -0.04 | 0.69 |
| 2 | 44,014,016 | rs10186876 | A | G | 0.64 | -0.16 | 2.7E-09 | -0.20 | 0.01 | 0.63 | | -0.25 | 0.004 | 0.22 | 0.06 | 0.81 | -0.07 | 0.66 | -0.25 | 0.003 |
| 2 | 70,476,715 | rs958685 | C | A | 0.52 | 0.15 | 2.8E-09 | 0.12 | 0.12 | 0.50 | | 0.13 | 0.12 | 0.53 | 0.15 | 0.44 | 0.20 | 0.21 | 0.11 | 0.16 |
| 6 | 32,493,090 | rs78325334 | C | T | 0.84 | 0.23 | 2.4E-09 | -- | -- | -- | | -- | -- | -- | -- | -- | -- | -- | -- | -- |
| 9 | 115,236,607 | rs72762373 | G | A | 0.03 | 0.42 | 4.9E-08 | 0.26 | 0.30 | 0.03 | | 0.41 | 0.11 | 0.01 | -1.36 | 0.21 | 0.42 | 0.41 | 0.20 | 0.45 |
| 10 | 102,367,414 | rs2273555 | G | A | 0.61 | 0.15 | 9.1E-09 | -0.01 | 0.85 | 0.60 | | -0.03 | 0.76 | 0.59 | 0.06 | 0.77 | -0.18 | 0.27 | 0.09 | 0.28 |
| 10 | 129,039,010 | rs374532236^*^ | T | C | 0.62 | -0.16 | 5.5E-09 | -0.25 | 0.003 | 0.63 | | -0.31 | 0.0003 | 0.76 | 0.14 | 0.55 | -0.26 | 0.12 | -0.18 | 0.03 |
| 11 | 74,644,478 | rs72979233 | A | G | 0.24 | -0.21 | 3.7E-12 | -0.18 | 0.06 | 0.24 | | -0.21 | 0.03 | 0.08 | 0.03 | 0.94 | -0.48 | 0.01 | 0.00 | 0.99 |
| 11 | 133,922,749 | rs34845616 | G | A | 0.25 | 0.17 | 1.7E-08 | 0.11 | 0.26 | 0.24 | | 0.06 | 0.55 | 0.05 | 0.66 | 0.13 | 0.22 | 0.28 | 0.05 | 0.60 |
| 12 | 14,911,061 | rs11614333 | C | T | 0.38 | -0.18 | 5.0E-11 | -0.14 | 0.08 | 0.37 | | -0.13 | 0.12 | 0.39 | -0.22 | 0.28 | -0.35 | 0.03 | -0.04 | 0.64 |
| 12 | 79,293,191 | rs10861798 | A | G | 0.57 | -0.14 | 4.3E-08 | -0.12 | 0.14 | 0.59 | | -0.08 | 0.33 | 0.92 | -0.59 | 0.10 | -0.36 | 0.03 | 0.00 | 0.97 |
| 17 | 46,147,574 | rs80103986^*^ | T | A | 0.81 | 0.20 | 1.8E-09 | 0.14 | 0,17 | 0.77 | | 0.13 | 0.19 | 0.95 | 0.50 | 0.26 | 0.22 | 0.28 | 0.12 | 0.26 |
| 17 | 48,583,930 | rs2288278 | G | A | 0.66 | 0.16 | 3.0E-09 | 0.20 | 0.02 | 0.66 | | 0.25 | 0.005 | 0.47 | 0.02 | 0.90 | 0.28 | 0.09 | 0.16 | 0.05 |
| 17 | 81,502,092 | rs6565586 | T | A | 0.25 | 0.17 | 2.2E-08 | 0.06 | 0.53 | 0.25 | | -0.01 | 0.95 | 0.23 | 0.35 | 0.12 | -0.03 | 0.86 | 0.11 | 0.22 |

EAF: Effect allele Frequency; Alt: Alternate (Effect) allele; Pos: Positions in build GRCh38; B: effect size, unit (kg); EA: European-Americans; AA: African-Americans

--: no result available (variant or proxies did not pass quality control in TOPMed)

* For rs374532236, proxy rs1556659 used in TOPMed EA (r2=0.98, 1000 Genomes); for rs80103986, proxy rs62063212 used in TOPMed EA (r2=0.98, 1000 Genomes) and AA (r2=1, 1000 Genomes)

**S10 Table.** Top association results from the whole genome sequence association analysis of *MEAN* handgrip strength (P<0.001 in at least one TOPMed analyses) among the 1,452 genome-wide significant variants from the UKBB handgrip strength GWAS [9]

|  |  |  | **UKBB (N=142,343)** | | | | | **TOPMed** | | | | | | | | | | | | |
| --- | --- | --- | --- | --- | --- | --- | --- | --- | --- | --- | --- | --- | --- | --- | --- | --- | --- | --- | --- | --- |
|  |  |  |  |  |  |  |  | **TOTAL (N=13,552)** | | | **EA (N=10,263)** | | | **AA (N=3,145)** | | | **MEN (N=4,878)** | | **WOMEN (N=8,674)** | |
| **Chr** | **Pos** | **rsid** | **Ref** | **Alt** | **EAF** | **B** | ***P*** | **B** | ***P*** | **EAF** | | **B** | ***P*** | **EAF** | **B** | ***P*** | **B** | ***P*** | **B** | ***P*** |
| 1 | 53,574,997 | rs10788958 | C | G | 0.65 | 0.16 | 1.3E-08 | 0.29 | 4.3E-04 | 0.63 | | 0.29 | 0.001 | 0.19 | 0.30 | 0.24 | 0.38 | 0.03 | 0.22 | 0.01 |
| 1 | 53,577,041 | rs2950250 | T | G | 0.66 | 0.16 | 5.0E-09 | 0.28 | 5.2E-04 | 0.65 | | 0.26 | 0.003 | 0.31 | 0.36 | 0.08 | 0.41 | 0.02 | 0.18 | 0.03 |
| 1 | 53,583,046 | rs2950252 | C | G | 0.66 | 0.16 | 9.8E-09 | 0.27 | 8.1E-04 | 0.65 | | 0.25 | 0.004 | 0.31 | 0.35 | 0.09 | 0.38 | 0.02 | 0.18 | 0.03 |
| 1 | 53,595,660 | rs7551844 | T | C | 0.66 | 0.17 | 1.1E-09 | 0.27 | 9.6E-04 | 0.65 | | 0.28 | 0.001 | 0.23 | 0.27 | 0.24 | 0.37 | 0.03 | 0.20 | 0.02 |
| 1 | 53,598,265 | rs11206176 | G | A | 0.66 | 0.17 | 1.3E-09 | 0.27 | 9.7E-04 | 0.65 | | 0.28 | 0.002 | 0.23 | 0.30 | 0.20 | 0.35 | 0.04 | 0.21 | 0.01 |
| 2 | 43,897,947 | rs4245797 | C | T | 0.40 | 0.15 | 4.3E-08 | 0.27 | 9.3E-04 | 0.42 | | 0.30 | 4.5E-04 | 0.77 | 0.13 | 0.59 | 0.29 | 0.08 | 0.24 | 0.003 |
| 2 | 43,945,305 | rs28394191 | A | C | 0.40 | 0.15 | 4.6E-08 | 0.22 | 0.005 | 0.41 | | 0.28 | 0.001 | 0.54 | -0.08 | 0.68 | 0.17 | 0.29 | 0.24 | 0.003 |
| 2 | 43,946,283 | rs7594526 | T | G | 0.40 | 0.15 | 3.6E-08 | 0.27 | 6.0E-04 | 0.41 | | 0.29 | 7.4E-04 | 0.64 | 0.15 | 0.46 | 0.27 | 0.10 | 0.25 | 0.002 |
| 10 | 129,036,434 | rs1556659 | C | T | 0.38 | 0.16 | 6.8E-09 | 0.24 | 0.003 | 0.37 | | 0.31 | 3.4E-04 | 0.24 | -0.14 | 0.55 | 0.26 | 0.12 | 0.18 | 0.03 |
| 10 | 129,036,519 | rs1015605 | G | A | 0.36 | 0.15 | 3.8E-08 | 0.24 | 0.003 | 0.36 | | 0.31 | 3.8E-04 | 0.24 | -0.13 | 0.58 | 0.26 | 0.12 | 0.18 | 0.03 |
| 17 | 48,551,644 | rs3826542 | G | A | 0.70 | 0.17 | 6.1E-09 | 0.28 | 8.4E-04 | 0.69 | | 0.33 | 3.2E-04 | 0.41 | 0.12 | 0.55 | 0.43 | 0.01 | 0.19 | 0.02 |
| 17 | 48,551,870 | rs890432 | G | A | 0.70 | 0.17 | 3.2E-09 | 0.28 | 9.3E-04 | 0.69 | | 0.33 | 3.9E-04 | 0.41 | 0.13 | 0.51 | 0.43 | 0.01 | 0.19 | 0.03 |
| 17 | 48,552,231 | rs2229304 | G | T | 0.70 | 0.16 | 7.9E-09 | 0.28 | 9.0E-04 | 0.69 | | 0.33 | 3.2E-04 | 0.42 | 0.11 | 0.58 | 0.44 | 0.01 | 0.19 | 0.03 |
| 17 | 48,586,488 | rs4793937 | G | A | 0.18 | -0.19 | 4.8E-08 | -0.47 | 3.8E-06 | 0.18 | | -0.47 | 1.6E-05 | 0.17 | -0.55 | 0.03 | -0.76 | 2.5E-04 | -0.31 | 0.003 |

EAF: Effect allele Frequency; Alt: Alternate (Effect) allele; Pos: Positions in build GRCh38; B: effect size, unit (kg); EA: European-Americans; AA: African-Americans

**S11** and **S12** **Tables** are available in a separate Excel document (S4 File).

**S13 Table.** Study-specific results for the low-frequency or common AA-specific associations detected using the whole genome sequence association analysis of *MEAN* handgrip strength in TOPMed

1. AA

|  |  |  |  |  | **HYPERGEN (N=1,696)** | | | **WHI (N=456)** | | | **CHS (N=536)** | | | **ARIC (N=457)** | | |
| --- | --- | --- | --- | --- | --- | --- | --- | --- | --- | --- | --- | --- | --- | --- | --- | --- |
| **Chr** | **Pos (build 38)** | **rsid** | **Ref** | **Alt** | **EAF** | **Beta** | ***P*** | **EAF** | **Beta** | ***P*** | **EAF** | **Beta** | ***P*** | **EAF** | **Beta** | ***P*** |
| 2 | 198,070,149 | rs74688411 | A | G | 0.02 | 3.56 | 0.003 | 0.01 | 4.60 | 0.02 | 0.02 | 2.76 | 0.06 | 0.03 | 4.70 | 0.002 |
| 5 | 76,919,524 | rs577776684 | C | T | 0.02 | -5.40 | 1.10E-05 | 0.01 | -0.01 | 0.99 | 0.01 | 2.50 | 0.21 | 0.01 | -6.45 | 0.01 |
| 8 | 30,273,968 | rs2958754 | G | A | 0.10 | -0.49 | 0.35 | 0.11 | -2.09 | 0.002 | 0.07 | -2.79 | 2.0E-04 | 0.10 | -0.45 | 0.57 |
| 10 | 85,774,936 | rs569475444 | G | C | 0.03 | -2.76 | 0.004 | 0.02 | 0.78 | 0.66 | 0.02 | -4.02 | 0.01 | 0.02 | 1.15 | 0.54 |
| 11 | 113,977,732 | rs182799368 | G | T | 0.01 | -3.11 | 0.02 | 0.01 | 0.72 | 0.73 | 0.02 | -3.68 | 0.02 | 0.02 | -4.84 | 0.02 |
| 14 | 96,043,941 | rs143569685 | T | A | 0.01 | -3.01 | 0.02 | 0.01 | -4.21 | 0.02 | 0.02 | -3.69 | 0.01 | 0.01 | -3.31 | 0.13 |

1. AA MEN

|  |  |  |  |  | **HYPERGEN (N=628)** | | | **WHI (N=0)** | | | **CHS (N=191)** | | | **ARIC (N=168)** | | |
| --- | --- | --- | --- | --- | --- | --- | --- | --- | --- | --- | --- | --- | --- | --- | --- | --- |
| **Chr** | **Pos (build 38)** | **rsid** | **Ref** | **Alt** | **EAF** | **Beta** | ***P*** | **EAF** | **Beta** | ***P*** | **EAF** | **Beta** | ***P*** | **EAF** | **Beta** | ***P*** |
| 2 | 198,070,149 | rs74688411 | A | G | 0.02 | 5.35 | 0.04 | -- | -- | -- | 0.01 | 8.95 | 0.02 | 0.03 | 6.23 | 0.03 |
| 5 | 76,919,524 | rs577776684 | C | T | 0.01 | -14.25 | 3.0E-06 | -- | -- | -- | -- | -- | -- | -- | -- | -- |
| 8 | 30,273,968 | rs2958754 | G | A | 0.09 | 0.59 | 0.60 | -- | -- | -- | 0.07 | -2.74 | 0.12 | 0.10 | 0.67 | 0.69 |
| 10 | 85,774,936 | rs569475444 | G | C | 0.03 | -8.52 | 6.5E-06 | -- | -- | -- | 0.01 | -13.92 | 2.6E-04 | 0.02 | -2.07 | 0.58 |
| 11 | 113,977,732 | rs182799368 | G | T | -- | -- | -- | -- | -- | -- | 0.02 | -10.66 | 0.003 | 0.03 | -7.90 | 0.01 |
| 14 | 96,043,941 | rs143569685 | T | A | -- | -- | -- | -- | -- | -- | -- | -- | -- | -- | -- | -- |

1. AA WOMEN

|  |  |  |  |  | **HYPERGEN (N=1,068)** | | | **WHI (N=456)** | | | **CHS (N=345)** | | | **ARIC (N=289)** | | |
| --- | --- | --- | --- | --- | --- | --- | --- | --- | --- | --- | --- | --- | --- | --- | --- | --- |
| **Chr** | **Pos (build 38)** | **rsid** | **Ref** | **Alt** | **EAF** | **Beta** | ***P*** | **EAF** | **Beta** | ***P*** | **EAF** | **Beta** | ***P*** | **EAF** | **Beta** | ***P*** |
| 2 | 198,070,149 | rs74688411 | A | G | 0.02 | 2.48 | 0.03 | 0.01 | 4.60 | 0.02 | 0.02 | 0.63 | 0.65 | 0.03 | 3.70 | 0.02 |
| 5 | 76,919,524 | rs577776684 | C | T | 0.02 | -2.54 | 0.02 | 0.01 | -0.01 | 0.99 | 0.01 | 4.10 | 0.02 | 0.01 | 0.81 | 0.77 |
| 8 | 30,273,968 | rs2958754 | G | A | 0.10 | -1.00 | 0.05 | 0.11 | -2.09 | 0.002 | 0.08 | -2.76 | 1.3E-04 | 0.11 | -1.04 | 0.21 |
| 10 | 85,774,936 | rs569475444 | G | C | 0.02 | 0.86 | 0.39 | 0.02 | 0.78 | 0.66 | 0.02 | 0.44 | 0.77 | 0.02 | 2.78 | 0.16 |
| 11 | 113,977,732 | rs182799368 | G | T | 0.01 | 0.73 | 0.59 | 0.01 | 0.72 | 0.73 | 0.02 | -0.77 | 0.62 | 0.01 | -0.53 | 0.85 |
| 14 | 96,043,941 | rs143569685 | T | A | 0.01 | -2.93 | 0.03 | 0.01 | -4.21 | 0.02 | 0.02 | -3.81 | 0.004 | 0.01 | -5.75 | 0.01 |

EAF: Effect allele Frequency; Alt: Alternate (Effect) allele; Pos: Positions in build GRCh38; Beta: effect size, unit (kg)

AA: Black/African-American

--: no result available (minor allele count less than 5)
