# Supplementary material for: Identification of novel and rare variants associated with handgrip strength using whole genome sequence data from the NHLBI Trans-Omics in Precision Medicine (TOPMed) Program
Source: PLoS One. 2021 Jul 2;16(7):e0253611. doi: 10.1371/journal.pone.0253611 (PMC8253404; doi:10.1371/journal.pone.0253611)
Supplement: Attachment — Submitted filename: Responses_to_Reviewers_041321.docx [file pone.0253611.s005.docx]

**Responses to the Reviewers' comments:**

We would like to thank the reviewers for their thoughtful comments and appreciation of our work. We have responded below, in blue, to all reviewers’ comments and revised the manuscript accordingly.

Review Comments to the Author

**Reviewer #1:** In this manuscript, Chloe Sarnowski and colleagues conducted a whole-genome sequence single-variant association and a gene-based rare/functional variant association studies to identify genomic regions associated with handgrip strength as a continuous variable. The authors discovered 10 loci from the single-variant analysis mostly in participants of AA and also identified one significant gene region from the gene-based analysis using EA sample. Although they used the high-quality sequence data, the sample size was fairly limited (N=13,552, even smaller in the stratified analyses) and the findings were not confirmed in an independent dataset (replication). The paper overall is well-written, and the authors well described and discussed the limitation of the study. Some minor issues could be addressed before publication.

Here are some things the authors could do to improve the clarity of the manuscript.

1. Abstract and Discussion: Please replace “15 new loci associated with handgrip strength” with “11 new loci …” since four gene regions from the gene-based test did not pass the significance level.

We have revised these sentences in the manuscript accordingly.

2. What's the rationale for adjusting for height in addition to BMI? Why different covariates were used in the UKBB sample (no sex interaction term)? Please explain in the Method section.

We have included in our association analyses the same set of covariates as was used in the previous published GWAS of handgrip strength (Willems et al, *Nat Commun*, 2017). Height is a proxy for hand size and BMI is capturing the component of handgrip strength related to body size.

We thank the reviewer for noticing the discrepancy between the set of covariates included in our TOPMed and UK Biobank analyses. We have tested the significance of the two additional interaction terms in a null model (interaction of age and BMI with sex) in the UK Biobank. As both were strongly associated with handgrip strength (see Table below), we have re-run the association analysis in the UK Biobank African-ancestry sample. We have revised the Supplementary Table with the replication results (now **Supplementary Table 8**) as well as the Method section describing the UK Biobank analyses.

Table 1: Association of covariates with handgrip strength in the UK Biobank African-ancestry sample

| **covariates** | **beta** | **pval** |
| --- | --- | --- |
| Age | 2.35 | **2.0E-16** |
| Age^2^ | 0.02 | 7.9E-01 |
| Height | 2.12 | **2.0E-16** |
| BMI | 0.16 | 7.1E-02 |
| SexMale | 7.84 | **2.0E-16** |
| pc2 | -0.43 | **6.1E-07** |
| pc3 | -0.62 | **1.0E-12** |
| pc4 | -0.94 | **2.0E-16** |
| pc7 | -0.43 | **6.8E-07** |
| pc15 | 0.21 | **1.7E-02** |
| Inter_agesex | -0.29 | **9.5E-04** |
| Inter_bmisex | 0.32 | **2.6E-04** |

3. Were there any newly identified loci from the TOPMed AA analysis that had a significant association in UKBB EA participants (trans-ancestry loci)?

The newly identified signals from the TOPMed AA analysis are specific to African-ancestry populations and do not exist or are very rare in European populations (see Table 2 below). Thus, they are not present in the Willems *et al*. EA handgrip strength GWAS summary statistics.

Table 2: Frequency of signals identified in the TOPMed African-ancestry handgrip strength association analysis in the 1000 Genomes (1kG) reference panel

| **rsids** | **Frequency in 1kG AFR** | **Frequency in 1kG EUR** |
| --- | --- | --- |
| rs74688411 | 0.02 | 0 |
| rs577776684 | 0.01 | 0 |
| rs377692678 | 0.006 | 0 |
| rs544430450 | 0.003 | 0 |
| rs2958754 | 0.12 | 0.002 |
| rs569475444 | 0.02 | 0 |
| rs189542078 | 0.008 | 0 |
| rs182799368 | 0.02 | 0 |
| rs143569685 | 0.02 | 0 |
| rs185725127 | 0.006 | 0 |

4. Please include the UKBB sample information in Table 1.

We thank the reviewer for this suggestion. We have modified **Table 1** and **Supplementary Tables 3 and 4** to include UKBB information and removed the descriptive Table of UKBB alone (previously **Supplementary Table 13**).

5. First paragraph of Result section: Did the authors actually test for handgrip strength differences by sex or race/ethnicities? Please provide a p value if the authors would like to use the term “lower”.

Following the reviewer’s suggestion, we performed formal t-tests to evaluate handgrip strength differences by sex and race/ethnicities.

*Women versus Men:*

***t = 104.9, df = 7270.3, p-value < 2.2e-16***

*EA versus AA:*

***t = -11.266, df = 4868.2, p-value < 2.2e-16***

However, as these differences could be confounded by different age and sex distributions in the EA and AA samples, we decided not to include these tests in the paper and to rephrase the first paragraph of the Result Section:

“Mean handgrip strength (SD) was equal to 23.5 kg (6.5) in women and 40.1 (9.9) in men. Mean age (SD) was 71 years (11) in EA participants and 60 years (18) in AA participants. Mean handgrip strength was equal to 28.9 kg (10.9) in EA participants and 31.6 kg (11.9) in AA participants.”

6. It would be helpful to include the phenotypic correlation between multiple handgrip traits in the manuscript (e.g. MEAN vs ONE).

We thank the reviewer for this suggestion. The overall handgrip strength phenotypic correlation between the *MEAN* and the *ONE* analysis was equal to 0.98. As several studies included in our analysis had only one exam where handgrip strength was available, we also calculated the phenotypic correlation by study. The handgrip strength phenotypic correlation between the *MEAN* and the *ONE* analysis was equal to 0.96, 0.95 and 0.93 for FHS, WHI and CHS respectively (the three studies for which multiple exams were available for handgrip strength). This information has now been added to the manuscript in the Methods section:

“The handgrip strength phenotypic correlation between the *MEAN* and the *ONE* analysis was equal to 0.96, 0.95 and 0.93 for FHS, WHI and CHS respectively”.

7. Table 3: Suggest this goes in Supplementary.

We have now moved **Table 3** to the Supplement (now **Supplementary Table 5**). We have added one footnote to Table 2 to indicate which results were significant in the sex-stratified analyses and referred to **Table 5** in **S1 Text**.

**Reviewer #2:**

“Leveraging handgrip strength measures and whole-genome sequences of six trans-ethnic studies, the authors associated 15 new loci with handgrip strength with rare and/or ethnicity-specific genetic variations. The statistical analyses were appropriately and rigorously performed using a set of advanced statistical methods. Their results support their conclusions. The manuscript has been well written. It is technically sound and reads interesting. For some potential improvements, I have a couple of comments/suggestions as below.

1. The participants used were from six study-reported ethnic groups, where African-Americans and Hispanics/Latinos have two and more ancestral populations. Some alleles are minor in one ancestral population but are major alleles in another ancestral population. It is unclear that how the authors defined “minor alleles”, i.e., in their principal component analyses (PCA) and relatedness estimates using the pooled genotypic data.

We thank the reviewer for this comment. The variant calling in TOPMed has been performed jointly across all studies. The genotype files used for PCA were coded in terms of the same reference allele for all studies. We did not attempt to define minor alleles in any population, as this step is unnecessary for PCA and relatedness calculations. The effect allele frequency (EAF) presented in the manuscript has been calculated in the whole TOPMed sample.

2. Participants from one ethnic group (i.e., European-Americans) would be unrelated to the participants from another ethnic group (i.e., African-Americans). Applying the pcrelate to pooled trans-ethnic study population would generate random relatedness estimates for numerous pairs of unrelated participants. It is unclear that how the authors avoid such random errors.

We would like to clarify that participants from multiple ethnic groups are not necessarily unrelated. For example, the children of two people who identify with different ethnic groups may identify with either or both of the parents’ groups and will be related to people from each group. We have observed many cases of relationships between participants in different studies in TOPMed, so accurately accounting for relatedness between all participants in the analysis, and not just within study or ethnic group, is necessary to get appropriately calibrated association test statistics. PC-Relate was actually designed precisely for the scenario of estimating relatedness in a sample of individuals with multiple ancestries. PC-Relate adjusts for population structure (ancestry) among sample individuals through the use of ancestry representative principal components (PCs) to provide accurate relatedness estimates due only to recent family (pedigree) structure. It reports kinship values at or near zero for any pair of participants who are unrelated (in the sense of recent pedigree structure), and it will not produce random relatedness estimates for unrelated pairs of participants.

3. For fine mapping in admixed populations, i.e., African-Americans and Hispanics/Latinos, adjusting for local ancestries proved a necessary and effective avenue to prevent false positives. Neglecting local ancestries could be one reason for the observed inflations in current results. It would be instructive to discuss or justify why not adjusting for local ancestries together with the global ancestries (PC1 – PC11).

We thank the reviewer for bringing up this point. We reviewed the literature on this topic (PMC3042179, PMC5079159, PMC5811405). These works indicate that local ancestry analysis is not required for variant discovery, but could be used to better inform follow-up analysis. Although we did not perform local ancestry analysis for this paper, it could be an avenue for future work to further investigate effects of the detected variants. We have added one sentence in the Discussion section:

“Finally, we have not considered in this paper local ancestry analysis that could help in the future to further investigate the effects of the detected variants.”

Also, we note that much of the literature on local ancestry assumes that SNP array data is used, so that LD patterns in the population studied have a strong effect on which variants are detected. Since our analysis is based on WGS, we think that local population stratification should not have biased the variant discovery.

4. The authors adjusted for a small number of selected covariates in their analyses. Showing their significant effects with p-values would be informative to justify why they should be adjusted for. Besides, the authors could have neglected certain important latent confounders due to limited data availability. Applying some method/package (i.e., BACON) could help control the confounding of latent confounders.

Following the reviewer’s suggestion, we are displaying in the Table below the significance of all covariates from the null model.

Table 3: Association of covariates with handgrip strength in the TOPMed sample for the three different models of handgrip strength

|  | **MEAN** | | **ONE** | | **ALL** | |
| --- | --- | --- | --- | --- | --- | --- |
| **covariates** | **beta** | **pval** | **beta** | **pval** | **beta** | **pval** |
| Age | -0.12 | **1.7E-03** | -0.05 | 1.8E-01 | -0.06 | **7.9E-02** |
| Age2 | -0.003 | **5.6E-39** | -0.004 | **9.5E-44** | -0.005 | **1.9E-112** |
| Height | 0.28 | **4.6E-231** | 0.28 | **9.8E-202** | 0.26 | **3.6E-210** |
| BMI | 0.16 | **1.2E-04** | 0.13 | **3.8E-03** | 0.34 | **5.4E-21** |
| SexMale | 21.90 | **3.6E-97** | 21.55 | **8.5E-86** | 22.84 | **5.8E-151** |
| Amish | 0.19 | 9.4E-01 | 0.09 | 9.7E-01 | 2.71 | 2.6E-01 |
| CHS | 2.67 | **4.4E-07** | 3.63 | **9.2E-11** | 5.89 | **1.4E-02** |
| FHS_gen3 | 0.49 | 4.7E-01 | 1.10 | 1.3E-01 | 0.61 | 8.0E-01 |
| FHS_offsp | -0.21 | 7.3E-01 | 0.49 | 4.7E-01 | 0.65 | 7.8E-01 |
| HYPERGEN | -3.45 | **8.8E-07** | -3.33 | **9.7E-06** | -3.23 | 1.7E-01 |
| WHI | -0.61 | 3.9E-01 | 0.35 | 6.4E-01 | 0.54 | 8.2E-01 |
| pc1 | -334.94 | **1.8E-25** | -410.89 | **1.4E-32** | -332.88 | **4.6E-24** |
| pc2 | 73.48 | 6.4E-01 | 44.05 | 7.9E-01 | 58.64 | 7.1E-01 |
| pc3 | -133.21 | 4.1E-01 | -143.35 | 4.1E-01 | -182.23 | 2.7E-01 |
| pc4 | 90.61 | 7.5E-01 | 103.89 | 7.4E-01 | 40.32 | 8.9E-01 |
| pc5 | 33.60 | 8.3E-01 | 43.37 | 8.0E-01 | 61.85 | 7.0E-01 |
| pc6 | -92.45 | 2.7E-01 | -125.57 | 1.6E-01 | -95.44 | 2.9E-01 |
| pc7 | -120.26 | 3.4E-01 | -117.25 | 3.9E-01 | -110.16 | 4.1E-01 |
| pc8 | -67.08 | 4.7E-01 | -66.19 | 5.1E-01 | -52.14 | 5.8E-01 |
| pc9 | -21.01 | 6.6E-01 | -24.99 | 6.2E-01 | -20.69 | 6.8E-01 |
| pc10 | -130.52 | **3.2E-03** | -124.85 | **8.5E-03** | -125.06 | **6.0E-03** |
| pc11 | 33.06 | 3.9E-01 | 18.98 | 6.3E-01 | 16.42 | 6.7E-01 |
| inter_agesex | 0.16 | **3.9E-43** | 0.15 | **2.4E-30** | 0.21 | **1.3E-112** |
| inter_bmisex | -0.05 | **4.8E-02** | -0.03 | 1.7E-01 | -0.13 | **1.9E-10** |
| inter_ARICsex | 0.49 | 1.8E-01 | 0.88 | **2.9E-02** | -0.42 | 2.3E-01 |
| inter_CHSsex | -0.21 | 5.5E-01 | -0.41 | 2.6E-01 | -1.31 | **2.7E-05** |

We also used the R package bacon to estimate bias and inflation corrected P-values for our main signals from the *MEAN* analysis, as suggested by the reviewer. All results passing the genome-wide threshold in our main analysis remained significant (see Table below).

Table 4: Corrected P-values for the 10 SNPs passing genome-wide significance in the TOPMed handgrip strength association analysis

| **Chr** | **Pos (build 38)** | **rsid** | **Ref** | **Alt** | ***P ALL*** | ***P MEN*** | ***P WOMEN*** |
| --- | --- | --- | --- | --- | --- | --- | --- |
| 2 | 198,070,149 | rs74688411 | A | G | 1.2E-09 |  |  |
| 5 | 76,919,524 | rs577776684 | C | T |  | 5.5E-10 |  |
| 5 | 174,630,802 | rs377692678 | G | A | 1.2E-09 |  |  |
| 7 | 95,524,137 | rs544430450 | G | A | 2.7E-09 |  |  |
| 8 | 30,273,968 | rs2958754 | G | A |  |  | 1.2E-09 |
| 10 | 85,774,936 | rs569475444 | G | C |  | 3.1E-10 |  |
| 10 | 119,692,415 | rs189542078 | C | T |  |  | 1.9E-09 |
| 11 | 113,977,732 | rs182799368 | G | T |  | 4.4E-10 |  |
| 14 | 96,043,941 | rs143569685 | T | A |  |  | 1.2E-09 |
| 18 | 71,404,509 | rs185725127 | A | C | 1.7E-10 |  | 2.9E-09 |

5. For the single marker association results, presenting 95% concentration intervals (CIs) of the genomic inflation coefficients at 25%, 50% and 75% quantiles would be informative to show how well the confounders were controlled. Without such CIs, it is hard to judge how confident that the confounders were well controlled.”

Following the reviewer’s suggestion, we calculated the genomic inflation factor lambda at the 25%, 50% and 75% quantiles for the *MEAN* analysis:

**25%** λ=1.078

**50%** λ=1.081

**75%** λ=1.092

It is not usual to calculate 95% confidence interval for the genomic inflation factor. We computed it for the median and the SE was very small: λ=1.081 95%CI: [1.080-1.082]. We expect the CI for the genomic inflation factor lambda at the 25% and 75% to be of similar range/magnitude.
