# Supplementary material for: Identification of novel and rare variants associated with handgrip strength using whole genome sequence data from the NHLBI Trans-Omics in Precision Medicine (TOPMed) Program
Source: PLoS One. 2021 Jul 2;16(7):e0253611. doi: 10.1371/journal.pone.0253611 (PMC8253404; doi:10.1371/journal.pone.0253611)
Supplement: Attachment — Submitted filename: Reviewers_Comments_II_v3.docx [file pone.0253611.s006.docx]

**Review Comments to the Author**

We have provided clarifications to all the reviewer’s questions or concerns, highlighted in blue below.

Reviewer #2: The authors inadequately addressed my comments as detailed below.

1. The participants used were from six study-reported ethnic groups, where African-Americans and Hispanics/Latinos have two and more ancestral populations. Some alleles are minor in one ancestral population but are major alleles in another ancestral population. It is unclear that how the authors defined “minor alleles”, i.e., in their principal component analyses (PCA) and relatedness estimates using the pooled genotypic data.

New Comment 1:

Replying this comment, the authors said they did not attempt to define minor alleles in any population. In their revision, however, the authors still used a “minor allele frequency (MAF)” threshold of 0.01 in their description of PCA and used “minor allele count” in Table 3. They should clarify these in some way, i.e., either replacing “minor allele” with a better phrase or clarify in what a sense they defined minor allele.

New response:

For PCA and relatedness estimates in TOPMed, genetic variants filtering included a minor allele frequency (MAF) threshold of 0.01. We have now clarified in the manuscript that the “minor” allele has been defined based on overall allele frequency in all of TOPMed:

“PCA and relatedness (IBD) estimates were made for 140,062 TOPMed samples using 638,486 SNVs passing QC, linkage disequilibrium (LD)-pruned from the genotypes with minimal depth >10× with a minor allele frequency (MAF) threshold of 0.01 (where “minor” allele is defined based on overall allele frequency in all of TOPMed), a missing call rate (MCR) threshold of 0.01 and a LD threshold of 0.32.“

The genotype files were coded using the same reference allele for all studies and ancestries. The effect allele frequency (EAF) in our manuscript Tables was estimated in the relevant group used in the analyses. The minor allele count (MAC) was derived based on the sample size (N) and the minor allele frequency (MAF) (i.e., MAC=2×N×MAF), where the MAF was defined as the EAF if EAF<0.50 and 1-EAF if EAF>=0.50. We provided clarifications in the Method section:

“The genotype files were coded in terms of the same reference allele for all studies and ancestries and the “minor” allele was defined based on the overall allele frequency in TOPMed.”

“The effect allele frequency (EAF) was estimated in the relevant group used in the analyses. The minor allele count (MAC) was derived based on the sample size (N) and the MAF (i.e., MAC=2×N×MAF), where the MAF was defined as the EAF if EAF<0.50 or 1-EAF if EAF>=0.50.”

2. Participants from one ethnic group (i.e., European-Americans) would be unrelated to the participants from another ethnic group (i.e., African-Americans). Applying the pcrelate to pooled trans-ethnic study population would generate random relatedness estimates for numerous pairs of unrelated participants. It is unclear that how the authors avoid such random errors.

New comment 2:

In their response to this comment, the authors stated that the pcrelate reported kinship values “near” zero for pairs of unrelated participants**.** Such near-zero estimates are very common and what I meant by “random relatedness”. The package they used might have used some arbitrary truncation threshold to determine pairs of unrelated persons. Explicitly stating the parameters used can be informative for the readers.

New response:

Based on the reviewer’s suggestion, we have now defined in the Method section the threshold used to define the unrelated samples in PC-AiR. We have also clarified the method, which uses an "unrelated" threshold only for calculating PCs, not for generating the kinship matrix:

“PC-AiR partitions samples into ‘unrelated’ and ‘related’ sets based on genotypes and then performs PCA on the ‘unrelated’ set, and finally projects PC values for the ‘related’ set. A threshold of 2^(-9/2) as inferred by the KING ibdseg algorithm was used for defining the ‘unrelated’ set, meaning that a set of samples less related than 3^rd^ degree was used to calculate the PCs. Relatedness was estimated using the GENESIS R package 'pcrelate' function, which estimates kinship coefficients and IBD sharing probabilities conditional on ancestry. The resulting relatedness matrix includes pairwise kinship estimates for all samples, not just those used to calculate PCs.”

3. For fine mapping in admixed populations, i.e., African-Americans and Hispanics/Latinos, adjusting for local ancestries proved a necessary and effective avenue to prevent false positives. Neglecting local ancestries could be one reason for the observed inflations in current results. It would be instructive to discuss or justify why not adjusting for local ancestries together with the global ancestries (PC1 – PC11).

New comment 3:

To reply this comment, the authors reviewed several papers on local-ancestry adjustments (PMC3042179, PMC5079159, PMC5811405). Such papers actually proved the necessity of local-ancestry adjustments for fine mapping in admixed populations. In particular, for WGS data, it is instructive to consider local ancestries in addition to global ancestries for rare variant association mapping, see Sci Rep. 2019 Apr 1;9(1):5458; PMC6443736, Nature Genetics volume 44, pages243–246(2012), and Nature Genetics volume 53, pages195–204(2021).
Moreover, the averages of accurate genome-wide local ancestry proportions represent global ancestries much more accurately than do the major pcair PCs. By the authors’ Table 3, only PC1 and PC10 appeared significantly relevant. Even if I did not see the joint distributions of the major pcair PCs, I am frankly not sure if the pcair PCs well represent ancestries, see also my New Comment 4. When applying for the GENESIS package, tuning parameters must be carefully selected to determine the unrelated sets and the relatives set. I did not see the values and the justification.

New response:

The reviewer mentioned that the cited papers on local ancestry adjustments proved the necessity of local-ancestry adjustments for fine mapping in admixed populations. We acknowledged in our previous response that local ancestry analysis could be used to better inform follow-up analysis but is not required for variant discovery. We did not perform fine-mapping analysis in this paper, but we mentioned in the Discussion that further analyses such as local ancestry analysis could help to further investigate effects of the detected variants.

The LABST method, mentioned and developed by the reviewer, “only currently works for a recent admixture of two ancestral populations with different genetic architectures (i.e., distinct causal allele frequencies and/or effects).” While we agree that it could be instructive to consider local ancestries in addition to global ancestries for rare variant association mapping, the extension of this method for multi-way admixtures such as Hispanic and Latino Americans is outside the scope of this study. In our manuscript, we complemented our rare variant analyses performed in the pooled TOPMed sample with analyses performed separately in each ancestry with adequate sample size (EA and AA) and we observed consistent results. Only one suggestive gene was detected in gene-based tests in the pooled sample and not in the ancestry-specific analyses (because the lead variant was too frequent in AA to be included in the test).

We have added two sentences in the manuscript:

“We performed analyses in the pooled sample as well as stratified by ancestry, as rare variant aggregation tests can be underpowered for identifying rare variant associations in admixed populations.”

“Finally, we have not considered in this paper local ancestry analysis that could help in the future to further investigate the effects of the detected variants and ancestry-gene interaction that could boost statistical power of rare variant association mapping in admixed populations.”

To demonstrate that PCs well represent ancestries in TOPMed, we refer the reviewer to a figure in the flagship TOPMed paper (Taliun et al., 2021) showing the PCs color-coded by population group defined according to race, ancestry and/or ethnic information provided by the study participants and/or by study investigators according to study inclusion criteria:

We have edited the Method section accordingly:

“After reviewing the PCA plots as well as the PC-SNP correlation plots, we determined that PCs 1-11 detected ancestry among the TOPMed freeze 8 samples, and included PCs 1-11 in all analysis models, as recommended by the TOPMed Data Coordinating Center (for an example from an earlier TOPMed data freeze, see Extended Data Figure 1 in Taliun et al. 2021).“

The comment regarding the parameter used to define unrelated participants was addressed above (comment 2).

4. The authors adjusted for a small number of selected covariates in their analyses. Showing their significant effects with p-values would be informative to justify why they should be adjusted for. Besides, the authors could have neglected certain important latent confounders due to limited data availability. Applying some method/package (i.e., BACON) could help control the confounding of latent confounders.

New comment 4:

The authors provided Table 4 to show the handgrip-covariates associations under 3 distinct models. Multiple covariates, especially PC2, …, PC9, and PC11, appeared to be insignificant under each of the 3 models. Assuming the major PCs well represent ancestries – sufficiently capturing both between- and within-subpopulation structure. Jointly adjusting for study IDs and PCs might be unnecessary or even problematic, as indicated by the insignificance of multiple study IDs and PCs. Some PC plots stratified by study IDs might be informative to demonstrate the connections between PCs and study IDs.

Latent confounders would be common, and it is great to see BACON corrected P-values for the 10 SNPs passing genome-wide significance threshold. But I did not see any details of the adopted BACON parameters or the Q-Q plots of the BACON corrected p-values. The default parameters of the BACON package are often inappropriate and should be tuned carefully. See my New Comment 5.

New response:

We have provided below PCs plots for the first 3 PCs, stratified by study IDs (Figure 1), to show that, for each study, participants have a range of PC values. It is common practice when multiple studies are combined in an analysis to adjust for study as well as for principal components of genetic ancestry. The former accounts for any differences that are due to differing study design. If different studies have different mixes of ancestries, then adjustment for study also partially adjusts for ancestry. However, including PCs to adjust for the residual population structure in the studies is always recommended. Including covariates that are not significantly associated with the trait does not affect the power of the test of association between the genetic variant and the quantitative trait.

Figure 1: PCs plots (PC1 vs PC2; PC1 vs PC3; PC2 vs PC3) color-coded by study.

The BACON R package has been developed and evaluated for epigenetic or transcriptomic analyses, and all but one paper that we found citing the tool have used it in this context. We could find no evidence that it is widely used in the context of genetic analyses, and we did not find any recommendation on how to tune the parameters in the original paper (PMID: 28129774) or in a paper including genetic data that has used it, from the same group of authors/consortium (PMID: 30082726). As our study does not have severe inflation, and we have been cautious in the interpretation of our results, we do not believe that including BACON corrected p-values improves the manuscript.

5. For the single marker association results, presenting 95% concentration intervals (CIs) of the genomic inflation coefficients at 25%, 50% and 75% quantiles would be informative to show how well the confounders were controlled. Without such CIs, it is hard to judge how confident that the confounders were well controlled.

New Comment 5:

The authors appeared to misunderstand the above comment and derived λ=1.081 as the inflation factor for the median with 95% confidence interval [1.080-1.082]. Per this result, I am 95% confident the true inflation factor is larger than 1 since the lower confidence limit is larger than 1, aka, showing significant inflation.

A concentration interval is different from a confidence interval. They have different definitions and computation formulae. The number of effective tests plays critical roles on the computation of the concentration interval for a genomic inflation factor. Let their analysis be valid and let the proportion of the true association signals be much smaller than 0.25. Given the very large number of effective tests, the genomic inflation factors at the 25%, 50% and 75% quantiles should be much closer to 1 than the authors showed. In other words, the concentration intervals are very narrower with lower limits < 1 and upper limits > 1.

We thank the reviewer for the clarification. We were not familiar with the concept of a concentration interval. We found it in only two papers (PMID: 26458888; PMID: 30931973), in the context of simulations under the null hypothesis of no associations, to assess type I error for a method.

We note again that our genomic inflation factor computed at the median is not extreme, and within the range of many published GWAS of densely typed variants with mixed ancestries (for example PMC6785182). The commonly used rule of thumb is that genomic inflation factors <=1.1 are not considered concerning (<https://doi.org/10.1016/B978-0-12-813073-5.00018-6>). We have carefully accounted for both population structure via fixed effects using the PCs and cryptic relatedness through the random effect with the genetic relatedness matrix covariance structure, as is standard practice to avoid confounding. We note that confounders other than ancestry and cryptic relatedness are rare in genetic association studies.

Yang et al (PMID: 21407268) showed that the expected value of the genomic inflation factor is expected to be >1 even when there is no population structure (or it is properly accounted for), and is a function of sample size, LD structure, number of causal variants (polygenicity) and heritability of the trait under study. As $\lambda_{GC}$=1.08 in our study, and we have been cautious in the interpretation of our results, we do not feel that further analyses to further clarify the inflation are warranted.
